# Effect of the Dynamic Response of a Side-Wall Pressure Measurement System on Determining the Pressure Step Signal in a Shock Tube Using a Time-of-Flight Method

**DOI:** 10.3390/s22062103

**Published:** 2022-03-09

**Authors:** Andrej Svete, Francisco Javier Hernández Castro, Jože Kutin

**Affiliations:** Laboratory of Measurements in Process Engineering, Faculty of Mechanical Engineering, University of Ljubljana, Aškerčeva Cesta 6, SI-1000 Ljubljana, Slovenia; javier.hernandez-castro@fs.uni-lj.si (F.J.H.C.); joze.kutin@fs.uni-lj.si (J.K.)

**Keywords:** time-varying pressure, primary calibration method, diaphragmless shock tube, time-of-flight method, shock wave velocity, piezoelectric pressure measurement system, uncertainty analysis

## Abstract

Technological progress demands accurate measurements of rapidly changing pressures. This, in turn, requires the use of dynamically calibrated pressure meters. The shock tube enables the dynamic characterization by applying an almost ideal pressure step change to the pressure sensor under calibration. This paper evaluates the effect of the dynamic response of a side-wall pressure measurement system on the detection of shock wave passage times over the side-wall pressure sensors installed along the shock tube. Furthermore, it evaluates this effect on the reference pressure step signal determined at the end-wall of the driven section using a time-of-flight method. To determine the errors in the detection of the shock front passage times over the centers of the side-wall sensors, a physical model for simulating the dynamic response of the complete measurement chain to the passage of the shock wave was developed. Due to the fact that the use of the physical model requires information about the effective diameter of the pressure sensor, special attention was paid to determining the effective diameter of the side-wall pressure sensors installed along the shock tube. The results show that the relative systematic errors in the pressure step amplitude at the end-wall of the shock tube due to the errors in the detection of the shock front passage times over the side-wall pressure sensors are less than 0.0003%. On the other hand, the systematic errors in the phase lag of the end-wall pressure signal in the calibration frequency range appropriate for high-frequency dynamic pressure applications are up to a few tens of degrees. Since the target phase measurement uncertainty of the pressure sensors used in high-frequency dynamic pressure applications is only a few degrees, the corrections for the systematic errors in the detection of the shock front passage times over the side-wall pressure sensors with the use of the developed physical dynamic model are, therefore, necessary when performing dynamic calibrations of pressure sensors with a shock tube.

## 1. Introduction

The development of different industrial sectors has brought an urgent need for pressure sensors with suitable dynamic properties. In the automotive industry, accurate measurements of the time-varying pressure in the combustion chambers and the exhaust systems are important for improvement of the fuel economy and reduction of the emissions. Measurements of time-varying pressure are also vital in the steam and gas turbines in power plants in order to find the sources of losses and to determine the efficiencies of turbomachines. In manufacturing processes, e.g., injection moulding, accurate measurements of time-varying pressure are important for better process control and, therefore, for more efficient use of materials and energy. The highest requirements come from the aerospace industry, where the changing pressures with frequencies up to a few tens of kHz must be accurately measured. The importance of the accurate measurements of the time-varying pressure in the aerospace industry is evident from numerous studies. In [1], time-varying pressure was measured to exploit the effects of wakes from the upstream blade rows to control loss generation and, therefore, develop the new generation of ultra-high-lift low-pressure turbines. In [2], the importance of the time-varying pressure measurements for the determination of the combustion chamber entrance conditions, and, therefore, the performance of the entire propulsion system, is shown. In [3], the experimental and numerical investigations of the variation of the surface pressure were compared to understand the physical mechanism of the resistance to combustor back pressure. In [4], experiments were carried out to investigate the pressure fluctuations in the base region of a missile, which can induce an aerodynamic excitation leading to structural failure and stability problems. In [5], the pressure fluctuations that affect the buzz phenomenon in a supersonic inlet were experimentally investigated. The measurements of the surface pressure fluctuations were also required during the development of the new Space Launcher VEGA-C in order to prevent their effect on structural vibrations that are potentially dangerous for the payload and for other components of the launcher [6]. A study presented in [7] focused on the measurements of pressure oscillations of a practical core-engine annular combustor during the start up. The study in [8] showed the dominant role of pressure fluctuation on the aero-optical distortions, which are important for the development of airborne optical systems, i.e., missiles with optical seekers, airborne telescopes, airborne free-space communication systems and airborne laser weapon systems.

Metrological traceability to the International System of Units (SI) requires the development of appropriate primary standards for dynamic measurements of pressure [9,10,11,12,13,14,15,16]. The aperiodic shock tube pressure generators show a potential to provide SI-traceable dynamic calibrations for pressure sensors [17,18,19,20,21]. The reference pressure step signal generated at the end-wall of the driven section is defined by the pressure step amplitude and the arrival time of the initial shock front at the end-wall of the driven section. The amplitude of the generated pressure step at the end-wall of the driven section can be determined using traceable measurements of the shock wave velocity at the end-wall *V_wall_* and the initial absolute pressure *P*_1_ and temperature *T*_1_ of the gas in the driven section as [22,23]:

(1) $$\Delta P=2P_{1}\frac{\gamma_{1}}{\left(\gamma_{1}^{2}-1\right)}\left(M_{wall}^{2}-1\right)\left(\frac{M_{wall}^{2}\left(3\gamma_{1}-1\right)+3-\gamma_{1}}{M_{wall}^{2}+\frac{2}{\left(\gamma_{1}-1\right)}}\right),$$

where $\gamma_{1}$ is the adiabatic index, $M_{wall}=V_{wall}/a_{1}$ is the shock wave Mach number, $a_{1}=\sqrt{\gamma_{1}R_{1}T_{1}}$ is the speed of sound and *R*_1_ is the specific gas constant. The velocity of the shock wave generated in the shock tube attenuates along the driven section due to the thermal-viscous effects [24,25,26,27,28,29]. Therefore, *V_wall_* is usually extrapolated from the velocity distribution along the driven section determined using a time-of-flight (TOF) method. This method determines the shock wave velocity along the driven section using the times of the shock wave passages over the side-wall pressure sensors *t_i_* with the centers at locations *x_i_* along the driven section [30,31,32,33,34]. Considering the quadratic model for the variation of the shock wave velocity *V*(*x*) = *ax*^2^ + *bx* + *c*, four side-wall pressure sensors are required to obtain the constants *a*, *b* and *c* by solving the system of three equations [34]:

(2) $$t_{i+1}-t_{i}=\int_{x_{i}}^{x_{i+1}}\frac{dx}{V\left(x\right)},$$

where *i* = 1–3. The arrival time of the shock front at the end-wall is furthermore determined as:

(3) $$t_{wall}=t_{4}+\int_{x_{4}}^{x_{wall}}\frac{dx}{V\left(x\right)},$$

where *x_wall_* is the location of the end-wall.

The most important uncertainty component of the pressure step amplitude given by Equation (1) is associated with the end-wall shock wave velocity *V_wall_*, which typically represents more than 90% of the uncertainty of the predicted pressure step. In [33] we estimated that for the lowest observed driver pressure the uncertainty contribution of the shock wave velocity represented approximately 99%, while, at the highest observed driver pressure represented approximately 93% of the uncertainty of the predicted pressure step. After upgrading the shock tube with an additional side-wall pressure sensor installed near the end of the tube, which enabled us to describe the nonlinear decelerations of the normal shock waves along the tube, the uncertainty contribution of the shock wave velocity still represented 96% of the combined uncertainty for the lowest observed driver pressure and 97% for the highest observed driver pressure [34]. The fact that the uncertainty of the shock wave velocity represents a major component of the uncertainty of the pressure step has been confirmed also by other authors. In [35], the uncertainty contribution of the shock wave velocity without considering the uncertainty related to the deceleration of the shock waves was estimated to be approximately 80%, while, in [36], the same authors estimated the uncertainty contribution of the shock wave velocity, by considering also the uncertainty related to the deceleration of the shock waves, to be approximately 95%.

The uncertainties of the end-wall shock wave velocity *V_wall_* and the arrival time of the shock front *t_wall_* include the uncertainty of the extrapolation model, the uncertainty of the location of the end-wall, the uncertainties of the locations of the side-wall pressure sensors and the uncertainties of the detected times of the shock wave passages over these locations. The latter strongly depend on the dynamic response of the side-wall pressure measurement system (PMS). To the best of the knowledge of the authors of this paper, until now, the effects of non-ideal dynamic response of the side-wall pressure measurement system to the passage of the shock wave have not been studied. A better understanding of these effects can lead to possible corrections for the systematic errors and can, therefore, reduce the uncertainty of the pressure step signal determined at the end-wall of the driven section using the TOF method. In order to determine these systematic errors, a physical mathematical model that considers modelling the pressure input signal due to the transverse shock wave passage over the side-wall pressure sensors and the frequency response function of the complete measurement chain is required.

This paper evaluates the effect of the dynamic response of the side-wall PMS on the detection of the times of the shock wave passages over the side-wall pressure sensors. Furthermore, it evaluates its effect on the amplitude and the initial time of the pressure step at the end-wall determined using the TOF method. The experimental setup of the developed diaphragmless shock tube is presented in Section 2. Moreover, in Section 2, the amplitudes of the pressure steps at the end-wall of the developed diaphragmless shock tube are determined for different conditions. To determine the errors in the detection of the times of the shock wave passages over the centers of the side-wall sensors, a physical model for simulating the dynamic response of the PMS to the passage of the shock wave was developed. The model, which is presented in Section 3, considers the modelling of the pressure input signal due to the shock wave passage over the side-wall pressure sensors and the frequency response function of the complete pressure measurement chain, comprising a pressure sensor, a charge amplifier and a digitizer. In setting up the physical dynamic model, special attention was paid to determining the effective diameter of the pressure sensor, which is a key parameter that affects the course of the pressure input signal. Due to the design of the pressure sensor diaphragm mounting, the effective diameter of the sensing part of the sensor is generally smaller than the physical external diameter of the sensor and, therefore, must be evaluated experimentally. In Section 4, the errors in the detection of the shock front passage times over the side-wall pressure sensors and their contribution to the uncertainty of the generated end-wall pressure step signals are presented and discussed. The conclusions are drawn in Section 5.

## 2. Shock Tube Experimental Setup

**Figure 1:**
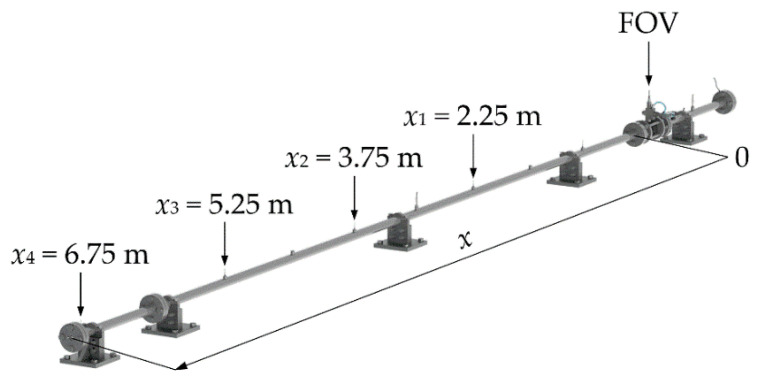
Schematic view of the diaphragmless shock tube experimental setup.

The study of the effect of the dynamic response of the side-wall PMS was realized on the developed diaphragmless shock tube of inner diameter of 40 mm with implemented fast-opening valve (FOV; ISTA, KB-40-100), see Figure 1. The construction of the diaphragmless shock tube and the test procedure are presented in detail in [33,34]. In this paper, we analysed the results of the tests performed with dry nitrogen 5.0 at the atmospheric initial driven pressure *P*_1_ and at the initial driver gauge pressures *P*_4,*g*_ from 4 MPa to 10 MPa.

**Figure 2:**
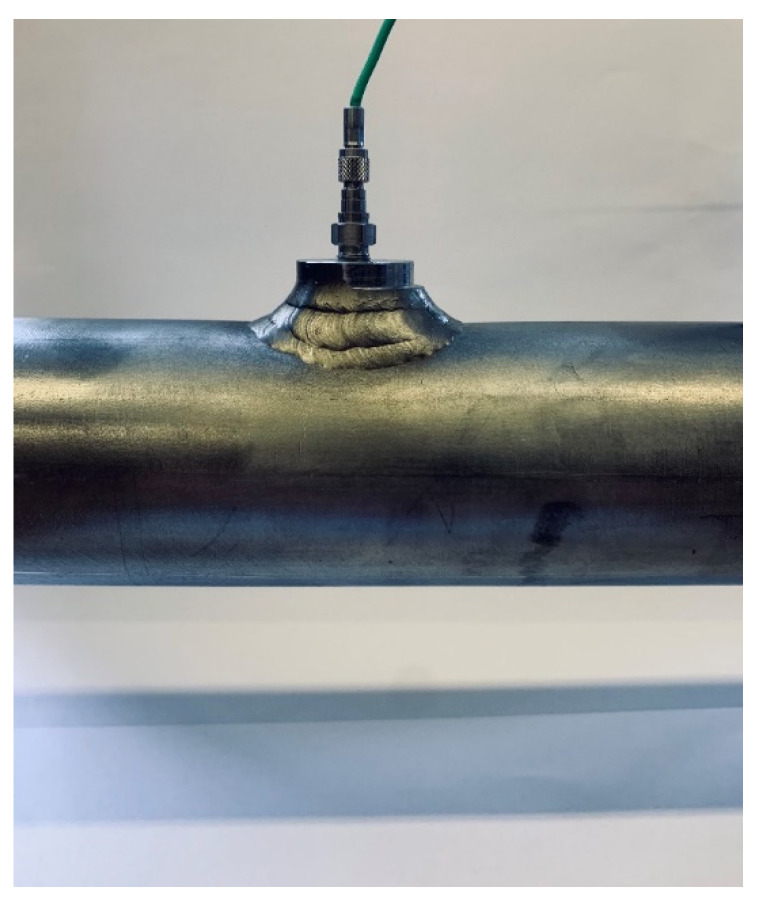
Closer view of the installed side-wall pressure sensor.

The supersonic velocity of the generated shock waves along the driven section was determined by solving the system of three Equation (2), where the measured times of the shock front passages over four identical side-wall piezoelectric pressure sensors (Kistler, 603CAA) that are flush mounted in the side wall of the driven section and their locations (denoted as *x*_1_, *x*_2_, *x*_3_ and *x*_4_ in Figure 1) were considered. A closer view of one of the installed side-wall pressure sensors is shown in Figure 2. The first side-wall pressure sensor was installed 2.25 m downstream of the FOV, where the shock wave reaches the developed conditions for the initial driver gauge pressures of 4 MPa or higher [34]. The separation distance of the adjacent pressure sensors *x_i_*_+1_ − *x_i_* for *i* = 1 and 2 is 1.5 m, for *i* = 3 it is 1.5019 m and *x_wall_* − *x*_4_ = 0.0684 m. The output signals of the side-wall pressure sensors are connected in series to the same input of the charge amplifier (Kistler, 5018A, integrated 2nd order low-pass Butterworth filter with a cut-off frequency of 200 kHz (−3 dB)). The group voltage output signal *U*(*t*) is, furthermore, acquired with a digitizer (National Instruments, NI 9775, sampling frequency 20 MHz, integrated 6th order low-pass analog Bessel filter with a cut-off frequency of 13.9 MHz (−3 dB)). The times of the shock wave passages over the centers of the side-wall pressure sensors were determined as the times at which the group voltage output signal gradients reach their local maxima. The local maximum gradients in the signal were determined as local maxima in the voltage output signal transformed by the mean-shift-based (MSB) method, which calculates the differences between the sum of the adjacent short signal segments of the same width that are time-shifted between each other by the width of the segment [34]:

(4) $$U_{msb}\left(nT_{s}+WT_{s}\right)=\sum_{n}^{n+W}U\left(nT_{s}+WT_{s}\right)-\sum_{n}^{n+W}U\left(nT_{s}\right),$$

where *T_s_* is the sampling period, *W* = 50 is the number of samples of the averaged signal segments and *n* = 0 … *N* − 1 − 2*W*, where *N* is the total number of samples in the output signal. The signal acquisition and processing are realized in the LabVIEW programming environment.

**Figure 3:**
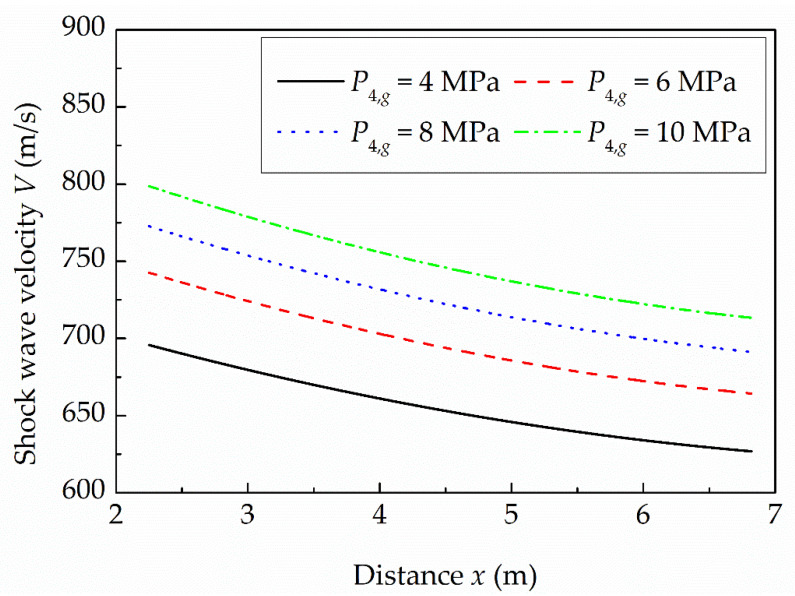
Measured shock wave velocities along the driven section for different initial driver gauge pressures.

Figure 3 presents the shock wave velocities along the driven section obtained for different initial driver gauge pressures *P*_4,*g*_. From the figure, it is clear that, due to the thermal-viscous effects, the velocity of the generated shock wave at the lowest initial driver gauge pressure of 4 MPa decreases from approximately 696 m/s at the position of the first side-wall pressure down to approximately 627 m/s at the end-wall, while, for the highest initial driver gauge pressure of 10 MPa, the velocity decreases from approximately 799 m/s down to 713 m/s. The average obtained functional dependencies of the velocity along the driven section are *V*(*x*) = 1.74*x*^2^ − 30.96*x* + 757.22 for *P*_4,*g*_ = 4 MPa, *V*(*x*) = 1.97*x*^2^ − 34.90*x* + 811.36 for *P*_4,*g*_ = 6 MPa, *V*(*x*) = 1.98*x*^2^ − 35.68*x* + 843.34 for *P*_4,*g*_ = 8 MPa and *V*(*x*) = 1.97*x*^2^ − 36.70*x* + 871.73 for *P*_4,*g*_ = 10 MPa. Therefore, the amplitude of the generated pressure step at the end-wall determined using Equation (1) increases from approximately 0.89 MPa to 1.42 MPa when increasing the initial driver gauge pressure from 4 MPa to 10 MPa.

## 3. Physical Dynamic Model of the Side-Wall PMS

**Figure 4:**
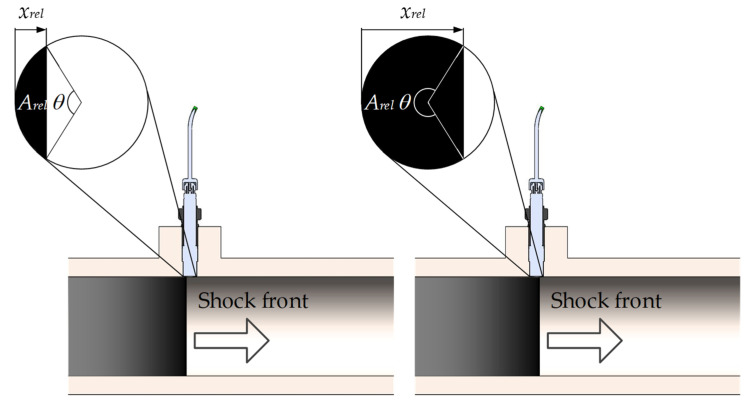
Relative portion of the sensor diaphragm’s effective area excited by the shock wave at different moments of its passage.

The pressure sensor installed in the side wall of the shock tube is subjected to the change of pressure due to transverse movement of the shock wave. When the shock wave passes over the diaphragm of the pressure sensor, the integral effect of the shock wave pressure on the pressure sensor is proportional to the size of the sensor diaphragm’s effective area covered by the shock wave. Thus, the pressure signal with the amplitude normalized between 0 and 1 can be defined by the relative portion of the sensor diaphragm’s effective area excited by the shock during its passage. Assuming a side-wall pressure sensor with a circular diaphragm with an effective diameter *D* that has the same sensitivity at all points and the shock wave velocity *V*, which can be assumed constant across the whole sensor’s diaphragm due to the fact that the effective diameter is negligibly small in comparison to the changes of the shock wave velocity along the shock tube, the normalized pressure input signal due to the shock wave passage can be written as:

(5) $$p\left(t\right)=\left\{ \begin{array}{ll} 0, & x_{rel}\left(t\right)\leq 0\\ A_{rel}\left(t\right), & 0<x_{rel}\left(t\right)<1\\ 1, & x_{rel}\left(t\right)\geq 1 \end{array}\right.,$$

where:

(6) $$x_{rel}\left(t\right)=\frac{V\cdot t}{D},$$

and the relative portion of the sensor diaphragm’s effective area excited by the shock wave passage, see Figure 4, is:

(7) $$A_{rel}\left(t\right)=\frac{\theta \left(t\right)-\sin\theta \left(t\right)}{2\pi },$$

with θ(t) representing the central angle in radians:

(8) $$\theta \left(t\right)=2\mathrm{acos}\left(1-2x_{rel}\left(t\right)\right).$$

The given pressure input signal is normalized to have values *p*(*t*) between 0 and 1, and *t* = 0 corresponds to the time when the shock front reaches the effective area of the sensor diaphragm.

The normalized voltage output signal of the PMS u(t) is determined as an inverse Fourier transform of the product of Fourier transform of the normalized pressure input signal p(t) and the normalized frequency response function (FRF) of the complete piezoelectric PMS under consideration $H_{PMS}\left(\omega \right).$ To apply the Fourier transform on the normalized pressure input signal p(t), which has unequal values at the interval endpoints, the signal has first to be converted into a duration-limited signal with equal endpoints $p^{*}\left(t\right)$ by using, e.g., the Gans–Nahman technique [37,38]:

(9) $$p^{*}\left(t\right)=\left\{ \begin{array}{ll} p\left(t\right), & 0\leq t\leq T\\ 1-p\left(t-T\right), & T<t\leq 2T \end{array}\right.,$$

which satisfies $p^{*}\left(0\right)=p^{*}\left(2T\right)=0,$ where *T* is the time period of the truncated signal.

The normalized PMS voltage output signal can, therefore, be determined as:

(10) $$u\left(t\right)={ℑ}^{-1}\left(ℑ\left(p^{*}\left(t\right)\right)H_{PMS}\left(\omega \right)\right).$$

**Figure 5:**
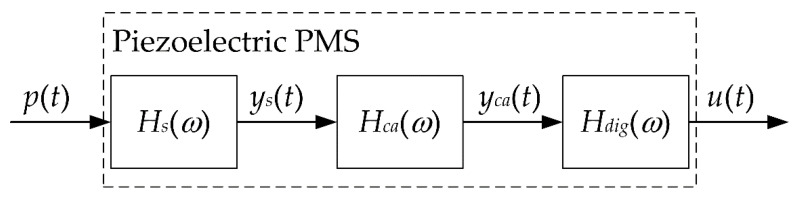
Components of the piezoelectric PMS.

All the components of the piezoelectric PMS are modelled as linear, time-invariant dynamic systems. Therefore, $H_{PMS}\left(\omega \right)$ equals the product of the FRFs of the pressure sensor $H_{s}\left(\omega \right),$ the charge amplifier $H_{ca}\left(\omega \right)$ and the digitizer $H_{dig}\left(\omega \right),$ see Figure 5. The pressure sensor is modelled as a second-order dynamic system with the FRF [39,40,41]:

(11) $$H_{s}\left(\omega \right)=\frac{1}{1-\frac{\omega^{2}}{\omega_{n}^{2}}+i2\xi \frac{\omega }{\omega_{n}}},$$

where the undamped natural frequency $f_{n}=\omega_{n}/2\pi$ and the damping ratio ξ of the piezoelectric pressure sensor under consideration were estimated in [34] to be 341.5 kHz and 0.0047, respectively. The FRF of the charge amplifier is [42]:

(12) $$H_{ca}\left(\omega \right)=\frac{1}{1-\frac{\omega^{2}}{\omega_{c,ca}^{2}}+1.4142i\frac{\omega }{\omega_{c,ca}}},$$

where the charge amplifier cut-off frequency $f_{c,ca}=\omega_{c,ca}/2\pi$ is 200 kHz, and the FRF of the digitizer is [42]:

(13) $$H_{dig}\left(\omega \right)=\frac{1}{\prod_{i}^{3}\left(1-b_{i}\frac{\omega^{2}}{\omega_{c,dig}^{2}}+a_{i}i\frac{\omega }{\omega_{c,dig}}\right)},$$

where *a*_1_ = 1.2217, *a*_2_ = 0.9686, *a*_3_ = 0.5131, *b*_1_ = 0.3887, *b*_2_ = 0.3505, *b*_3_ = 0.2756 and the digitizer cut-off frequency $f_{c,dig}=\omega_{c,dig}/2\pi$ is 13.9 MHz.

### Determination of the Effective Diameter of the Side-Wall Pressure Sensors

**Figure 6:**
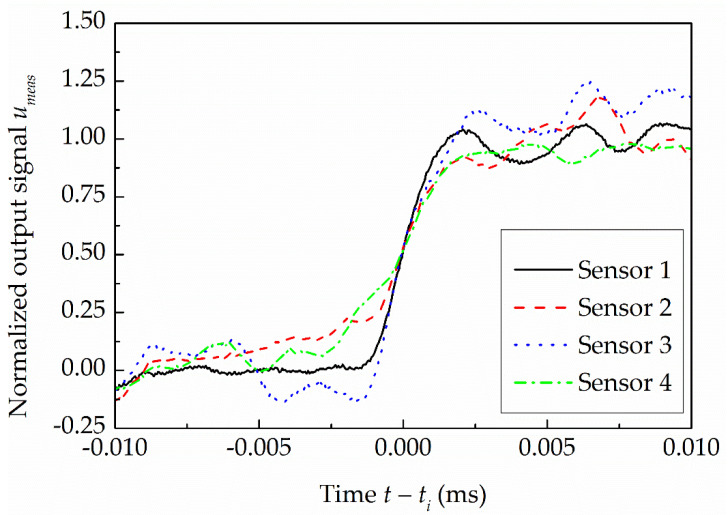
Normalized measured responses of the four identical side-wall pressure sensors installed along the driven section at *P*_4,*g*_ = 10 MPa.

The use of the physical model presented in the previous subsection requires information about the effective diameter of the pressure sensor. This parameter was determined based on the best fit between the normalized measured output signals of the side-wall PMS and the theoretical output signals obtained with the physical model, where, in the physical model, the shock wave velocities determined at the locations of the side-wall sensors *V_i_* were considered. The measured output signal of the side-wall pressure sensor was normalized with respect to the theoretical normalized output signal so that both signals had zero initial values and equal values at the detected passage time of the shock front:

(14) $$u_{meas}\left(t\right)=\frac{U\left(t\right)-U_{0}}{U\left(t_{i}\right)-U_{0}}u_{theor}\left(t_{i}\right),$$

where U(t) is the measured PMS’s group voltage output signal, $U_{0}$ is its average value prior to the sensor’s response to the shock wave passage (the averaging window has a length of 10 × 10^−3^ ms and ends at $t=D_{s}/2V_{i}$ prior to the determined passage time of the shock front, where *D_s_* = 5.55 mm is the physical external diameter of the piezoelectric pressure sensor specified by its manufacturer), $U\left(t_{i}\right)$ and $u_{theor}\left(t_{i}\right)$ are the values of the measured and normalized theoretical voltage output signal at the passage time of the shock front determined by applying the MSB method to the output signals, respectively. Figure 6 shows the normalized measured responses for all four identical side-wall pressure sensors at *P*_4,*g*_ = 10 MPa. From the figure, it is clear that, due to the fact that the output signals of all four pressure sensors are connected to the charge amplifier in series, the group voltage output signal prior to the response of the second, third and fourth sensors installed downstream of the FOV to the shock wave passage is greatly affected by the phenomenon behind the shock wave that, at that time, excites the upstream pressure sensors. These effects are also seen in larger oscillations of the signal prior to the response of the second, third and fourth sensors to the shock wave passage, which makes it difficult to determine $U_{0}$ for these three sensors. Therefore, the second, third and fourth side-wall pressure sensors installed downstream of the FOV, which are identical to the first side-wall pressure sensor, were excluded from further analyses in determining the side-wall pressure sensor’s effective diameter.

**Figure 7:**
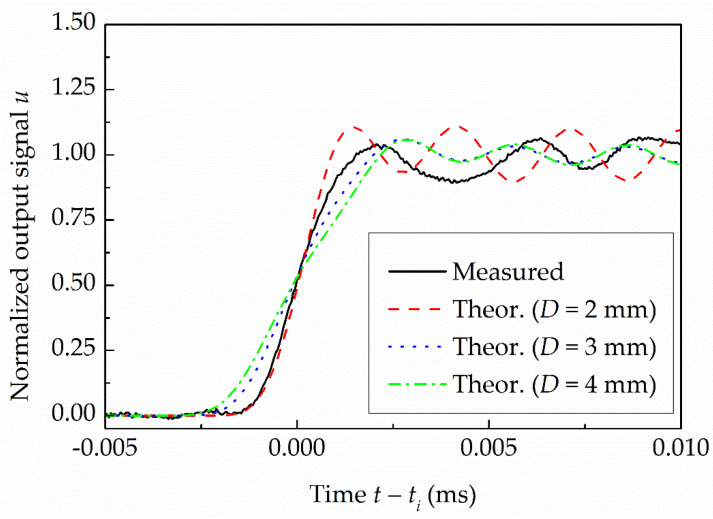
Normalized measured response of the first side-wall pressure sensor in comparison to the theoretical responses at *P*_4,*g*_ = 10 MPa.

Figure 7 shows the measured response of the first sensor installed downstream of the FOV at *P*_4,*g*_ = 10 MPa and the theoretical responses of the pressure sensors with diameters of 2 mm, 3 mm and 4 mm. As would be expected, the slope of the PMS’s output signals increases by decreasing the sensor’s diameter. From the figure, it is also clear that the slope of the theoretical response of the pressure sensor with a diameter between 2 mm and 3 mm would best resemble the slope of the actual measured response of the sensor to the shock wave passage.

**Figure 8:**
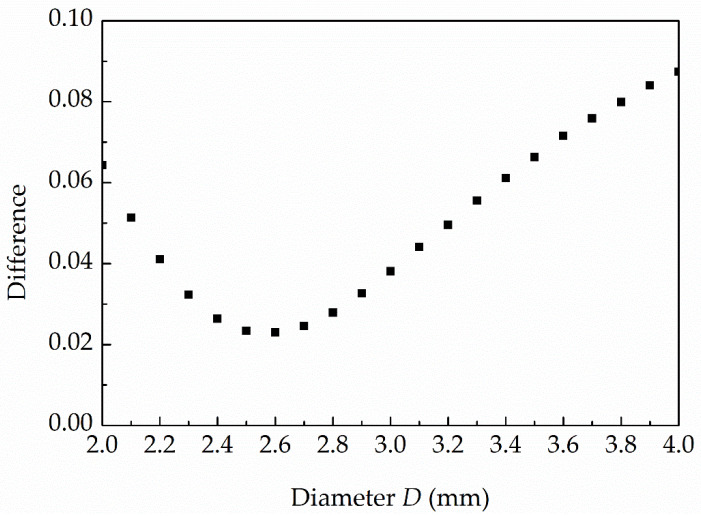
Average differences between the normalized measured and theoretical output signals obtained for different effective diameters of the side-wall pressure sensor.

To determine the effective diameter of the pressure sensor more accurately, the differences between the normalized measured responses obtained with the first side-wall pressure sensor from all the repeated measurements for all four initial driver pressures and the theoretical responses for different sensor’s effective diameters were calculated. The absolute values of the differences were determined in the range from 0.15 to 0.85 of the normalized output signals to avoid the effects of any additional oscillations in the measured PMS signal before and after the response of the sensors to the passage of the shock front that are seen in Figure 7. Figure 8 presents the average differences for the diameters from 2 mm to 4 mm with a step of 0.1 mm. The results show that the smallest average difference is obtained for the diameter of 2.6 mm, which determines the effective diameter of the four identical side-wall pressure sensors installed in the developed diaphragmless shock tube.

## 4. Results and Discussion

**Figure 9:**
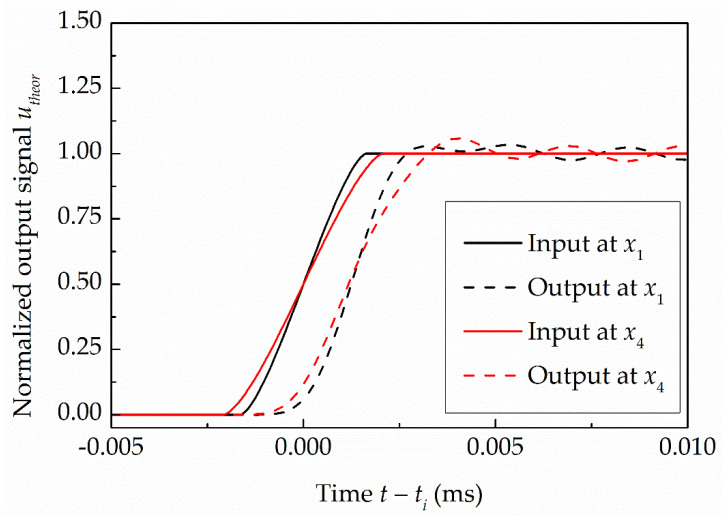
Normalized simulated input and output signals of the PMS at the location of the first side-wall pressure sensor at *P*_4,*g*_ = 10 MPa and fourth sensor at *P*_4,*g*_ = 4 MPa.

The errors in the detection of the shock front passage times over the side-wall pressure sensors were determined as the differences between the detected times of the shock front passages determined by applying the MSB method to the simulated output signals of the side-wall PMS and the passage times of the shock wave over the centers of the sensors *t_i_* determined by the time at which normalized simulated input signals reach the value 0.5. In the physical model, the shock wave velocities determined at the locations of the side-wall sensors and the effective diameter of the pressure sensor of 2.6 mm, as determined in Section 3, were considered. Figure 9 presents the effect of different shock wave velocities at different locations of identical pressure sensors along the driven section of the shock tube on the sensors’ input and output signals. The graph shows the signals for the first sensor at an initial driver gauge pressure of 10 MPa and the fourth sensor at an initial driver gauge pressure of 4 MPa, where the highest and the lowest observed shock wave velocities occur, respectively. It is clear that, by increasing the shock wave velocity, the slopes of the PMS’s input and output signals increase. Due to the fact that the component part of the PMS is also the pressure sensor, which is an underdamped second-order dynamic system, the response of the PMS to a relatively rapid pressure change shows an oscillation at the damped natural frequency of the pressure sensor. From Figure 9, it is also evident that the differences between the shock front passage times over the centers of the sensors and the times of the corresponding normalized output signals reaching a value of 0.5 are similar for both cases. Table 1 shows the errors in the detection of the shock front passage times over the centers of four side-wall pressure sensors *ε_i_*, where *i* = 1 to 4. The results for different generated shock wave velocities and different locations of the side-wall pressure sensors show that the time delays due to the response of the side-wall PMS are between 1.271 × 10^−3^ ms for the fourth sensor at the lowest observed initial driver pressure and 1.285 × 10^−3^ ms for the first sensor at the highest observed initial driver pressure.

The errors in the amplitude ∆*P* and the initial time *t_wall_* of the pressure step determined at the end-wall due to the errors in the detection of the shock front passage times over the side-wall pressure sensors using the TOF method are evaluated in accordance with JCGM 100:2008 [43]:

(15) $$\varepsilon_{y}=\sum_{i=1}^{4}c_{i}\varepsilon_{i},$$

where *y* is ∆*P* or *t_wall_*, $c_{i}={\partial y/\partial t}_{i}$ are the sensitivity coefficients and *ε_i_* are the systematic errors in the detection of the shock front passage times over the centers of the side-wall pressure sensors. Table 2 shows the relative errors in the amplitudes $\varepsilon_{\Delta P,r}=\varepsilon_{\Delta P}/\Delta P$ and the errors in the initial times $\varepsilon_{t_{wall}}$ of the pressure step at the end-wall. The results show that the relative errors in the pressure step amplitudes due to the side-wall PMS dynamics increase, on average, with increasing pressure step amplitude but are less than 0.0003% for all the cases and are, therefore, negligible in comparison to the target amplitude measurement uncertainty in dynamic calibrations of pressure sensors used in high-frequency dynamic pressure applications, which is at the level of 1%. On the other hand, the errors in the initial times of the pressure steps at the end-wall are approximately the same values as the errors in the detection of the shock front passage times over the center of the last side-wall pressure sensor and are up to 1.278 × 10^−3^ ms. This implies that the dynamics of the last sensor installed downstream of the driven section; therefore, the error in the detection of the passage time of the shock front for this sensor presents the most important contribution to the errors in the initial time of the pressure step at the end-wall. Although the determined errors in the initial time of the pressure step at the end-wall might seem relatively small, they represent very significant errors in determining the phase frequency characteristics of the pressure sensors using a shock tube. In the calibration frequency range of 100 kHz, which is the frequency range appropriate for high-frequency dynamic pressure applications [41], they represent systematic phase errors $\varepsilon_{\phi }={\omega \varepsilon }_{t_{wall}}$ of up to 46° [34]. Since the target phase measurement uncertainty of the pressure sensors used in high-frequency dynamic pressure applications is only a few degrees, the corrections for the systematic errors in the detection of the shock front passage times over the side-wall pressure sensors are, therefore, necessary when performing dynamic calibrations of pressure sensors with a shock tube.

## 5. Conclusions

This paper evaluates the effect of the dynamic response of the side-wall PMS on the detection of the times of the shock wave passages over the side-wall pressure sensors installed along the diaphragmless shock tube and, therefore, on the pressure step signal determined at the end-wall of the driven section using the TOF method. The study was performed on the results of the tests using dry nitrogen at the atmospheric initial driven pressure and at the initial driver gauge pressures from 4 MPa to 10 MPa, which generates shock waves with velocities from approximately 627 m/s to 799 m/s.

The errors in the detection of the shock front passage times over the side-wall pressure sensors were determined by applying the MSB method to the output signal of the side-wall PMS simulated with the use of the developed physical dynamic model. The developed physical model considers the pressure input to be proportional to the passage area of the shock front across the circular sensor’s diaphragm and simulates the PMS’s response by considering the dynamics of the complete piezoelectric measurement chain. The results show that the errors in the detection of the shock front passage times over the centers of four side-wall pressure sensors for different generated shock wave velocities are up to 1.285 × 10^−3^ ms.

The relative errors in the pressure step amplitudes determined at the end-wall of the shock tube due to the errors in the detection of the shock front passage times over the side-wall pressure sensors are, therefore, less than 0.0003%. This means that the uncertainty contribution of the systematic errors in the shock front passage times using the TOF method is negligible in comparison to the target calibration uncertainty of the pressure sensors used in high-frequency dynamic pressure applications, which is at the level of 1%. The results also show that the error in the detection of the shock front passage time over the center of the last side-wall pressure sensor installed downstream of the driven section, which is up to 1.278 × 10^−3^ ms, is the major source of the error in the initial time of the pressure step at the end-wall. Due to the fact that, in the calibration frequency range appropriate for high-frequency dynamic pressure applications, the errors in the initial time of the generated pressure step represent the systematic phase errors of up to a few tens of degrees, the corrections for the systematic errors in the detection of the shock front passage times over the side-wall pressure sensors are, therefore, necessary when performing dynamic calibration of pressure sensors with the shock tube.

## Figures and Tables

**Table 1:** Errors in the detection of the shock front passage times over the centers of the side-wall pressure sensors.

*P*_4,*g*_ (MPa)	Repetition	*ε*_1_ (ms)	*ε*_2_ (ms)	*ε*_3_ (ms)	*ε*_4_ (ms)
4	1	0.001276	0.001274	0.001272	0.001271
	2	0.001276	0.001274	0.001272	0.001271
	3	0.001276	0.001274	0.001272	0.001271
	4	0.001276	0.001274	0.001272	0.001271
	5	0.001276	0.001274	0.001272	0.001271
	6	0.001277	0.001274	0.001272	0.001271
	7	0.001277	0.001274	0.001272	0.001271
	8	0.001277	0.001274	0.001272	0.001271
	9	0.001277	0.001274	0.001272	0.001271
	10	0.001277	0.001274	0.001272	0.001271
6	1	0.001280	0.001278	0.001275	0.001274
	2	0.001280	0.001278	0.001275	0.001274
	3	0.001280	0.001278	0.001275	0.001274
	4	0.001280	0.001278	0.001275	0.001274
	5	0.001280	0.001278	0.001275	0.001274
	6	0.001280	0.001278	0.001275	0.001274
	7	0.001280	0.001278	0.001275	0.001274
	8	0.001280	0.001278	0.001275	0.001274
	9	0.001280	0.001278	0.001275	0.001274
	10	0.001280	0.001278	0.001275	0.001274
8	1	0.001283	0.001280	0.001278	0.001276
	2	0.001283	0.001280	0.001278	0.001276
	3	0.001283	0.001280	0.001278	0.001276
	4	0.001283	0.001280	0.001278	0.001276
	5	0.001283	0.001280	0.001278	0.001276
	6	0.001283	0.001280	0.001278	0.001276
	7	0.001283	0.001280	0.001278	0.001276
	8	0.001283	0.001280	0.001278	0.001276
	9	0.001283	0.001280	0.001278	0.001276
	10	0.001283	0.001280	0.001278	0.001276
10	1	0.001284	0.001282	0.001280	0.001278
	2	0.001285	0.001282	0.001280	0.001278
	3	0.001285	0.001282	0.001280	0.001278
	4	0.001285	0.001282	0.001280	0.001278
	5	0.001285	0.001282	0.001279	0.001278
	6	0.001285	0.001282	0.001280	0.001278
	7	0.001285	0.001282	0.001280	0.001278
	8	0.001285	0.001282	0.001280	0.001278
	9	0.001285	0.001282	0.001280	0.001278
	10	0.001285	0.001282	0.001280	0.001278

**Table 2:** Amplitudes, relative errors in the amplitudes and errors in the initial times of the pressure steps generated at the end-wall.

*P*_4,*g*_ (MPa)	Repetition	∆*P* (MPa)	$\varepsilon_{\Delta P,r}$ (%)	$\varepsilon_{t_{wall}}$ (ms)
4	1	0.886	0.00016	0.001271
	2	0.884	0.00016	0.001271
	3	0.888	0.00013	0.001271
	4	0.886	0.00016	0.001271
	5	0.886	0.00016	0.001271
	6	0.887	0.00011	0.001271
	7	0.890	0.00011	0.001271
	8	0.890	0.00016	0.001271
	9	0.886	0.00014	0.001271
	10	0.888	0.00016	0.001271
6	1	1.102	0.00014	0.001274
	2	1.104	0.00014	0.001274
	3	1.108	0.00014	0.001274
	4	1.112	0.00017	0.001274
	5	1.108	0.00016	0.001274
	6	1.107	0.00020	0.001274
	7	1.105	0.00020	0.001274
	8	1.107	0.00017	0.001274
	9	1.107	0.00017	0.001274
	10	1.104	0.00020	0.001274
8	1	1.276	0.00021	0.001276
	2	1.282	0.00018	0.001276
	3	1.279	0.00020	0.001276
	4	1.280	0.00018	0.001276
	5	1.284	0.00020	0.001276
	6	1.279	0.00020	0.001276
	7	1.280	0.00017	0.001276
	8	1.289	0.00017	0.001276
	9	1.282	0.00018	0.001276
	10	1.288	0.00015	0.001276
10	1	1.430	0.00018	0.001278
	2	1.423	0.00018	0.001278
	3	1.424	0.00018	0.001278
	4	1.421	0.00022	0.001278
	5	1.416	0.00017	0.001278
	6	1.421	0.00022	0.001278
	7	1.413	0.00022	0.001278
	8	1.411	0.00022	0.001278
	9	1.422	0.00025	0.001278
	10	1.427	0.00022	0.001278

## References

[1] Hodson H.P., Howell R.J. (2005). The role of transition in high-lift low-pressure turbines for aeroengines. Prog. Aerosp. Sci..

[2] Gnani F., Zare-Behtash H., Knotis K. (2016). Pseudo-shock waves and their interactions in high-speed intakes. Prog. Aerosp. Sci..

[3] He Y., Huang H., Yu D. (2016). Investigation of boundary-layer ejecting for resistance to back pressure in an isolator. Aerosp. Sci. Technol..

[4] Viji M., Vikramaditya N.S., Verma S.B., Ali N., Thakur D.N. (2017). Characteristics of base pressure fluctuations of a typical missile configuration with a propulsive jet. Aerosp. Sci. Technol..

[5] Farahani M., Daliri A., Younsi J. (2019). Supersonic inlet buzz detection using pressure measurement on wind tunnel wall. Aerosp. Sci. Technol..

[6] Camussi R., Di Marco A., Stoica C., Bernardini M., Stella F., De Gregorio F., Paglia F., Romano L., Barbagallo D. (2020). Wind tunnel measurements of the surface pressure fluctuations on the new VEGA-C space launcher. Aerosp. Sci. Technol..

[7] Qin H., Wang W. (2021). Transient aerodynamic performances and pressure oscillations of a core engine combustor during start up. Aerosp. Sci. Technol..

[8] Ding H., Yi S., Xu Y., Zhao X. (2021). Recent developments in the aero-optical effects of high-speed optical apertures: From transonic to high-supersonic flows. Prog. Aerosp. Sci..

[9] Choi I.-M., Yang I., Woo S.-Y. (2013). High dynamic pressure standard based on the density change of the step pressure generator. Metrologia.

[10] Downes S., Knott A., Robinson I. (2014). Towards a shock tube method for the dynamic calibration of pressure sensors. Phil. Trans. R. Soc. A.

[11] Hanson E., Olson D.A., Liu H., Ahmed Z., Douglass K.O. (2018). Towards traceable transient pressure metrology. Metrologia.

[12] Sarraf C., Damion J.P. (2018). Dynamic pressure sensitivity determination with Mach number method. Meas. Sci. Technol..

[13] Durgut Y., Aydemir B., Bağcı E., Akşahin E., İnce A.T., Uslukılıç U. (2018). Development of dynamic calibration machine for pressure transducers. J. Phys. Conf. Ser..

[14] Salminen J., Saxholm S., Hämäläinen J., Högström R. (2020). Advances in traceable calibration of cylinder pressure transducers. Metrologia.

[15] Slanina O., Quabis S., Derksen S., Herbst J., Wynands R. (2020). Comparing the adiabatic and isothermal pressure dependence of the index of refraction in a drop-weight apparatus. Appl. Phys. B.

[16] Amer E., Wozniak M., Jönsson G., Arrhén F. (2021). Evaluation of shock tube retrofitted with fast-opening valve for dynamic pressure calibration. Sensors.

[17] Pain H.J., Rogers E.W.E. (1962). Shock waves in gases. Rep. Prog. Phys..

[18] Matthews C., Pennecchi F., Eichstädt S., Malengo A., Esward T., Smith I., Elster C., Knott A., Arrhén F., Lakka A. (2014). Mathematical modelling to support traceable dynamic calibration of pressure sensors. Metrologia.

[19] Yao Z., Wang Z., Wang C., Lv J. (2018). A fast estimation of shock wave pressure based on trend identification. Meas. Sci. Technol..

[20] Wang Z., Yao Z., Li C., Lv J., Fu H., Mourelatos Z.P. (2019). A new fluctuation assessment method for the step response signals of pressure sensors. Mech. Syst. Signal Process..

[21] Yao Z., Wang Z., Liu X., Wang C., Shang Z. (2021). An improved low-frequency noise reduction method in shock wave pressure measurement based on mode classification and recursion extraction. ISA Trans..

[22] Holder D.W., Schultz D.L. (1962). On the Flow in a Reflected-Shock Tunnel.

[23] Yao Z., Liu X., Wang C., Yang W. (2020). Improved traceable measurement of the reflected step pressure in shock tube with the compensation of shock wave attenuation. Aerosp. Sci. Technol..

[24] Curzon F.L., Phillips M.G.R. (1971). Low attenuation shock tube: Driving mechanism and diaphragm characteristics. Can. J. Phys..

[25] Petersen E.L., Hanson R.K. (2001). Nonideal effects behind reflected shock waves in a high-pressure shock tube. Shock Waves.

[26] Anderson J.D. (2003). Modern Compressible Flow: With Historical Perspective.

[27] Stotz I., Lamanna G., Hettrich H., Weigand B., Steelant J. (2008). Design of a double diaphragm shock tube for fluid disintegration studies. Rev. Sci. Instrum..

[28] Hong Z., Pang G.A., Vasu S.S., Davidson D.F., Hanson R.K. (2009). The use of driver inserts to reduce non-ideal pressure variations behind reflected shock waves. Shock Waves.

[29] Nativel D., Cooper S.P., Lipkowicz T., Fikri M., Petersen E.L., Schulz C. (2020). Impact of shock-tube facility-dependent effects on incident- and reflected-shock conditions over a wide range of pressures and Mach numbers. Combust. Flame.

[30] Zelan M., Arrhén F., Jarlemark P., Mollmyr O., Johansson H. (2015). Characterization of a fiber-optic pressure sensor in a shock tube system for dynamic calibrations. Metrologia.

[31] Diao K., Yao Z., Wang Z., Liu X., Wang C., Shang Z. (2020). Investigation of vibration effect on dynamic calibration of pressure sensors based on shock tube system. Measurement.

[32] Sembian S., Liverts M. (2020). On using converging shock waves for pressure amplification in shock tubes. Metrologia.

[33] Svete A., Kutin J. (2020). Characterization of a newly developed diaphragmless shock tube for the primary dynamic calibration of pressure meters. Metrologia.

[34] Svete A., Kutin J. (2022). Identifying the high-frequency response of a piezoelectric pressure measurement system using a shock tube primary method. Mech. Syst. Signal Process..

[35] Downes S., Knott A., Robinson I. Uncertainty Estimation of Shock Tube Pressure Steps. Proceedings of the IMEKO XXI World Congress.

[36] Knott A., Robinson I.A. (2019). Dynamic characterisation of pressure transducers using shock tube methods. Trans. Inst. Meas. Control.

[37] Gans W.L., Nahman N.S. (1982). Continuous and discrete Fourier transforms of steplike waveforms. IEEE Trans. Instrum. Meas..

[38] Cormack G.D., McMullin J.N., Blair D.A. (1991). Comments on “Continuous and discrete Fourier transforms of steplike waveforms”. IEEE Trans. Instrum. Meas..

[39] Schweppe J.L., Eichberger L.C., Muster D.F., Michaels E.L., Paskusz G.F. (1963). Methods for the Dynamic Calibration of Pressure Transducers.

[40] (2002). A Guide for the Dynamic Calibration of Pressure Transducers.

[41] Hjelmgren J. (2002). Dynamic Measurement of Pressure—A Literature Survey.

[42] Mancini R. (2002). Op Amps for Everyone.

[43] BIPM, IEC, IFCC, ILAC, ISO, IUPAC, IUPAP, OIML (2008). Evaluation of Measurement Data—Guide to the Expression of Uncertainty in Measurement.

